# Horizontal Transmission of Malignancy: In-Vivo Fusion of Human Lymphomas with Hamster Stroma Produces Tumors Retaining Human Genes and Lymphoid Pathology

**DOI:** 10.1371/journal.pone.0055324

**Published:** 2013-02-06

**Authors:** David M. Goldenberg, David V. Gold, Meiyu Loo, Donglin Liu, Chien-Hsing Chang, Elaine S. Jaffe

**Affiliations:** 1 Center for Molecular Medicine and Immunology, Garden State Cancer Center, Morris Plains, New Jersey, United States of America; 2 Immunomedics, Inc., Morris Plains, New Jersey, United States of America; 3 Laboratory of Pathology, Center for Cancer Research, NCI, NIH, Bethesda, Maryland, United States of America; University of Navarra, Center for Applied Medical Research, Spain

## Abstract

We report the in-vivo fusion of two Hodgkin lymphomas with golden hamster cheek pouch cells, resulting in serially-transplanted (over 5–6 years) GW-532 and GW-584 heterosynkaryon tumor cells displaying both human and hamster DNA (by FISH), lymphoma-like morphology, aggressive metastasis, and retention of 7 human genes (*CD74, CXCR4, CD19, CD20, CD71, CD79b*, and *VIM*) out of 24 tested by PCR. The prevalence of B-cell restricted genes (*CD19, CD20, and CD79b*) suggests that this uniform population may be the clonal initiating (malignant) cells of Hodgkin lymphoma, despite their not showing translation to their respective proteins by immunohistochemical analysis. This is believed to be the first report of in-vivo cell-cell fusion of human lymphoma and rodent host cells, and may be a method to disclose genes regulating both organoid and metastasis signatures, suggesting that the horizontal transfer of tumor DNA to adjacent stromal cells may be implicated in tumor heterogeneity and progression. The B-cell gene signature of the hybrid xenografts suggests that Hodgkin lymphoma, or its initiating cells, is a B-cell malignancy.

## Introduction

The horizontal transmission of cancer has involved viral, genetic, and cell-cell fusion mechanisms [1]–[8], which are not mutually exclusive. With the recent appreciation of the interdependency and crosstalk between a tumor and its stroma, or microenvironment, the nature and biology of a neoplasm in a different, adapted, microenvironment could elucidate the importance and modulation of the cancer cell's genome as it evolves and progresses in malignancy with a new stroma, vasculature, and immune setting. The current study involves the transplantation and continuous propagation, with progression to metastasis, of two human lymphomas in unconditioned, adult golden hamsters, with evidence that cell-cell fusion established continuous hybrid tumor lines retaining select human genes, the potential for dissemination, and morphological resemblance to the original human lymphomas.

The implications of this gene transfer from Hodgkin lymphoma cells to recipient rodent stromal cells are discussed in terms of its origin and pathogenesis as a B-cell neoplasm. We believe this is the first report of a Hodgkin lymphoma-derived interspecies heterosynkaryon formation in vivo, resulting in sustained malignant cell lines. Using a model of different species provides a means to distinguish each contributor's genes to the fused progeny, such as those of the human tumor from the animal's mesenchymal stroma.

## Materials and Methods

### Ethics Statement

Use of human tissue was conducted with the approval of the Research Committee of the Veterans Administration Hospital, Pittsburgh, PA, with written informed consent obtained from each of the research subjects. Animal studies were conducted with the approval, and under the guidelines of the Research Committee of the Veterans Administration Hospital, Pittsburgh, PA.

### Transplantation studies

**Figure 1:**
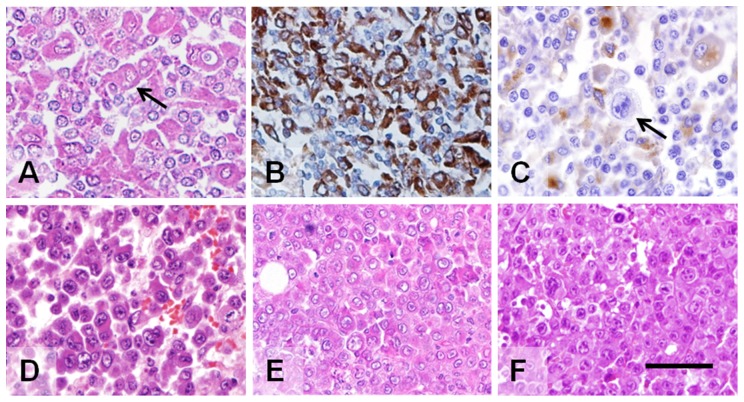
Microscopic morphology of the GW-532 human Hodgkin's lymphoma and xenografts derived by serial transplantation in the hamster cheek pouch. (A) Sections of the original axillary mass showed involvement by a lymphohistiocytic infiltrate composed of small lymphocytes, histiocytes, and plasma cells. Rare atypical mononuclear cells with features of Hodgkin cells were identified (arrow, Hodgkin-Reed-Sternberg cell). Immunohistochemical labeling of the original human tumor identified cells of (B) B/monocyte-cell origin (CD74^+^), and (C) macrophages (CD68^+^). A Hodgkin cell (arrow) is negative for CD68. Uniform morphological characteristics of the xenograft tumors from generations 2 (D), 25 (E), and 115 (F) were constant over time. The individual cells are large with vesicular chromatin and prominent central nucleoli. Occasional cells with binucleate or bilobed nuclei are present. Scale bar within image F corresponds to 100 µm for all images.

*GW-532 line*. Only limited samples of the original tumor remained from the patient's axillary mass that was biopsied. The tumor of this adult male extended from the axilla to the left chest wall and supraclavicular fossa. Sections of a left axillary lymph node showed effacement of the lymph node architecture and involvement by a lymphohistiocytic infiltrate composed of small lymphocytes, histiocytes, and plasma cells (Fig. 1A). Rare atypical mononuclear or binuclear cells with features of Hodgkin-Reed-Sternberg (HRS) cells were identified (arrow in Fig. 1A). Staining for CD74 (invariant chain) was positive, indicating the presence of B cells, monocytes and dendritic cells (Fig. 1B), while macrophages stained for CD68 (Fig. 1C). Histological features of this large axillary mass were reported previously, and were also diagnosed as Hodgkin lymphoma [9]. A suspension of these cells was injected into the cheek pouches of adult, unconditioned golden hamsters (*Mesocricetus auratus*), and then examined weekly for growth and gross spread. Resulting tumors were then excised and processed for histology, the animals dissected and examined for metastases [9]. An aliquot of tumor was further transplanted to the cheek pouch or other sites of adult, unconditioned hamsters for over 6 years.

#### GW-584 line

**Figure 2:**
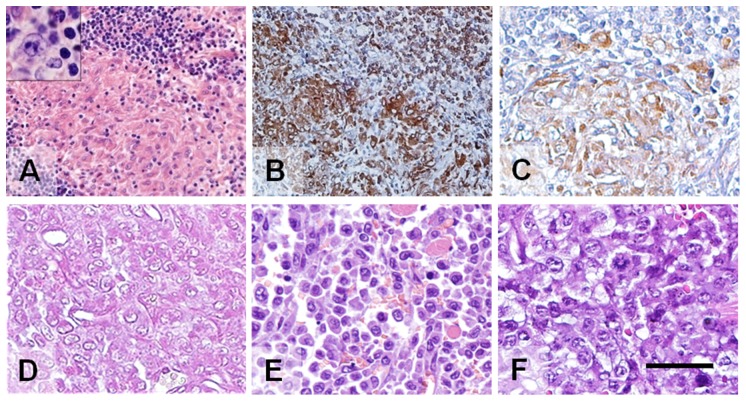
Microscopic morphology of GW-584 human mediastinal lymphoma and xenografts derived by serial transplantation in the hamster cheek pouch. (A) Sections of the original tumor showed effacement of the lymph node architecture. There was an extensive non-necrotizing granulomatous reaction with rare atypical mononuclear cells (inset) consistent with Hodgkin cells. (B) Immunohistochemical labeling of the original human tumor identified cells of (B) B/monocyte cell origin (CD74^+^), and (C) activated B cells and granuloma area (CD80^+^). Representative sections of xenografts obtained at transplant generations 1 (D), 28 (E), and 57 (F) show a constant uniform morphological appearance over time. The tumor is composed of large transformed lymphoid cells with vesicular nuclei and prominent nucleoli. Scale bar in F corresponds to 100 µm for images in C, D, E, and F, as well as the higher magnification inset within image A, and 400 µm for images in A and B.

A specimen of a mediastinal tumor of an adult male was available for grafting to the cheek pouches of adult, unconditioned golden hamsters. Only a small fragment of lymph node was available for histopathology, which showed effacement of the lymph node architecture. There was an extensive non-necrotizing granulomatous reaction (Fig. 2A). Rare atypical mononuclear cells consistent with Hodgkin cells were identified (Fig. 2A, insert), as well as HRS cells. Foci of necrosis and fibrosis also were present. The original human tumor was positive for CD74 (B cells, monocytes, and dendritic cells, Fig. 2B), CD68 (histiocytes), and CD80 (granuloma area, Fig. 2C) by immunohistochemistry. As in the prior transplant, resulting tumors were excised for histology and retransplantation, and the animals necropsied when metastasis was observed or suspected, or when they expired. This tumor line was propagated in adult, unconditioned hamsters, both in the cheek pouch and other sites, for over 5 years.

### Fluorescence in situ hybridization (FISH)

FITC-labeled human pancentromeric DNA probe was purchased from Cambio Ltd (Cambridge, UK). Cy3-labeled golden hamster probe was made in house with a PCR labeling kit (Jena Bioscience, Jena, Germany) as follows. The labeling reaction was carried out using a golden hamster centromeric satellite DNA (GenBank: AB185080.1) synthesized from Genscript (Piscataway, NJ) as template and two oligonucleotides as primers (forward: 5′-CACACACAGTGTTGTAACGTTAGTT-3′; reverse: 5′-GTGTGTGAAACTCTGTGCTTAACTCC-3′) at conditions of 94°C, 30 sec; 58°C, 30 sec; 72°C, 1 min for 30 cycles. The labeled probe was purified with Centri·Sep spin columns (Princeton Separation, Princeton, NJ) and visualized in 1% agarose gel.

FISH experiments were performed as described previously [10], with some modifications. Briefly, tissue sections were deparaffinized in two changes of xylene, digested in 0.025% proteinase solution (Protease XXIV, P8038, Sigma) for 2–3 h at 45°C, and then dehydrated in gradient ethanol. After air-drying, 10 µL of FITC-labeled human probe and 2 µL of Cy3-labeled hamster probe were pre-mixed and applied to the sections, cover-slipped and sealed with rubber cement. Co-denaturation at 73°C for 5 min and hybridization at 37°C overnight were done on a ThermoBrite hot plate (StatSpin, Westwood, MA). The next day, the slides were washed in 2×saline-sodium citrate buffer (SSC, Life Technologies, NY) for 2 min at RT, 2×SSC/0.3% Nonidet P40 for 1 min at 70°C, 2×SSC/0.1% Nonidet P40 for 1 min at RT, and 2×SSC for 2 min at RT. Then the slides were dehydrated in gradient ethanol, air-dried, and mounted with an antifade solution containing the nuclear counterstain 4, 6-diamidino-2-phenylindole (VectaShield, Vector Laboratories, Burlingame, CA). Image acquisition and analyses were performed using an Olympus fluorescence microscope (Olympus, Tokyo, Japan) and Kodak camera system (Kodak, Tokyo, Japan). The positive control for human genomic DNA was the GW-39 human colonic carcinoma transplant in the hamster cheek pouch [11]. The species-specificity of the Cy3-labeled golden hamster probe was demonstrated by staining RPMI 1846 cells, a golden hamster melanotic melanoma cell line from ATCC (Manassas, VA), but not human tumor cells or Chinese hamster CHO cells.

### Preparation of DNA from cells for PCR

Human and hamster genomic DNAs were purified from human Raji and CHO cells (both from ATCC) as positive and negative controls for the PCR of human genes, respectively. DNA was extracted from 1×10^6^ cells using DNeasy Tissue Kit (Qiagen, Germantown, MD), according to the manufacturer's instructions.

### DNA preparations from paraffin-embedded tissues

Paraffin-embedded tissues of human and hamster normal cells and tumors, or GW-532 and GW-584 transplants, were cut in 4- to 5-µm sections, and after treatment with xylene to deparaffinize the sections, DNA was extracted from each section with the QIAamp DNA FFPE Tissue Kit (Qiagen, Germantown, MD), according to the manufacturer's instructions.

Polymerase chain reaction (PCR) was performed to identify human genes and Epstein-Barr virus (EBV). Gene primers were selected from the UniSTS database of NCBI. The primers and their sources are provided in Table S1 in the Supporting Information online. All primers were custom-made by Eurofins MWG Operon (Huntsville, AL).

Each PCR sample contained 1 µL of DNA, 2.5 µL of 10× PCR buffer, 2.5 µL of the respective primer pairs (20 µM each) and 5 units of AmpliTaq DNA polymerase (1 µL). The PCR was repeated for 50 cycles, each consisting of denaturation at 94°C for 30 sec, annealing at 58°C for 30 sec, and polymerization at 72°C for 30 sec. The amplified fragments were analyzed on 2% agarose gel (Fisher Scientific (Fairlawn, NJ). The 10× PCR buffer and the AmpliTaq DNA polymerase were purchased from Applied Biosystems (Foster City, CA). Control positive cells for human DNA were Raji lymphoma cells (ATCC) and control negative cells for human DNA were Chinese hamster ovary (CHO) cells (ATCC).

### Antibodies and immunohistochemistry (IHC)

Immunohistochemistry for 24 proteins of the genes tested was performed on both the primary tumor and several transplant generations, listed with the antibody sources in Table S2 in the Supporting Information. These were selected on the basis of expression in lymphomas or other human transplants that we have studied, and included the typical markers for Hodgkin and non-Hodgkin lymphomas (e.g., CD15, CD19, CD20, CD22, CD23, CD30, and vimentin), as well as for proliferating and stromal cells associated with such tumors (CD68, CD71, CD74, and CD80).

Formalin-fixed, paraffin-embedded specimens were cut to 4-µm sections on superfrost plus adhesive slides (Thermo Scientific, Waltham, MA) and deparaffinized by routine methods. IHC staining was performed both with and without antigen retrieval by placing the slides in Target Retrieval Solution, pH 9.0 (Dako North America, Carpinteria, CA), and heating in a water bath at 95°C for 30 min, then cooling to room temperature. Primary antibodies, along with the appropriate species nonbinding controls, were then used at concentrations ranging from 1 to 10 µg/mL. An appropriate species-specific ABC Vectastain kit (Vector Laboratories, Burlingham, CA) was used as per the manufacturer's instructions for labeling tissues. For interpretation, a positive reaction was considered to be staining >5% of the appropriate tissue/cells. Murine irrelevant myeloma antibody (P3-X63-Ag8.653) from ATCC served as a negative control.

## Results

### Transplantation and histopathology

#### GW-532

**Figure 3:**
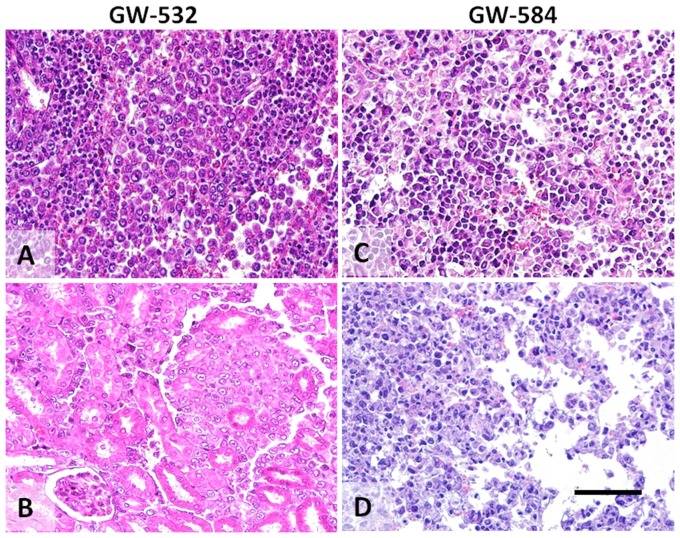
Xenografted tumors were highly aggressive in the hamster, with evidence of widespread metastatic disease. (A) and (B) show metastases in the hamster spleen and kidney, respectively, from a GW-532, generation-2, cheek pouch tumor. (C) and (D) show metastases in the hamster spleen and lung, respectively, from a GW-584, generation-3, cheek pouch tumor. Metastatic tumors show similar cytological appearance to the cheek pouch xenograft tumors. Scale bar in image D corresponds to 200 µm for all images.

Xenografts of the GW-532 tumor over many passages of its 6-year history were examined, including generation 115 that represented an almost 3-year serial propagation (Fig. 1D, E, and F). The morphological appearance was uniform and constant over time. The individual cells were large with vesicular chromatin and prominent central nucleoli resembling mononuclear Hodgkin and binucleate with rare bilobed nuclei similar to HRS cells (not shown), and had abundant eosinophilic cytoplasm. Spontaneous metastases to all major organs and regional lymph nodes with a similar cytological appearance were identified in many transplants, as early as 17 days after the initial hamster passage; representative metastases from the hamster cheek pouch tumor to the spleen and kidney are shown in Fig. 3A and B.

#### GW-584

Various transplant passages were examined during this tumor's 5-year history, indicating the morphology was constant since the very first transplant (Fig. 2D, E, and F). The 57^th^ passage (Fig. 2F), representing serial propagation for about 15 months, was morphologically identical to the initial transplant (Fig. 2D), with the tumor composed of large transformed lymphoid cells with vesicular nuclei and prominent nucleoli. Tumor cells invaded skeletal muscle and adipose tissue of the hamster cheek pouch. Spontaneous metastases from the cheek pouch tumors (or from other transplantation sites, such as subcutaneous and intracerebral) to all major organs and regional lymph nodes were identified (not shown). Evidence of metastasis was observed as early as 21 days of the very first cheek-pouch transplant, and had a similar cytological appearance to the cheek pouch tumor. Representative examples of metastases from the cheek pouch to the spleen and lungs are shown in Fig. 3C and D. The cytological features are similar to those of the xenografts of the GW-532 tumor cell line.

### Species-specific FISH DNA probes

**Figure 4:**
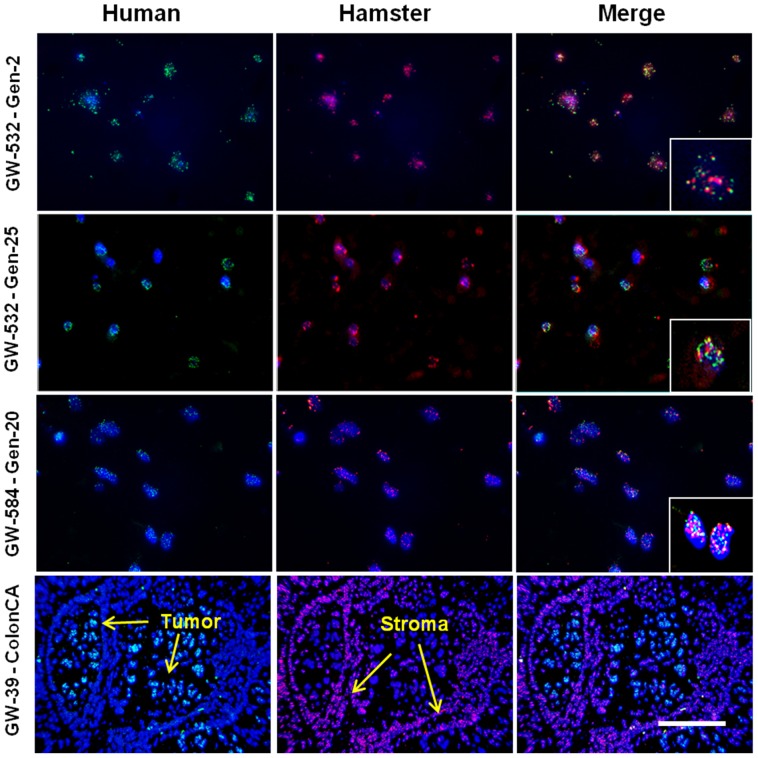
FISH of GW-532, early and late generations, and GW-584 late generation tumor cells was performed with the human (green) and golden hamster (red) pancentromeric probes to evaluate the co-existence of species-specific genomic material within these cells. A human colon carcinoma (GW-39) grown in the hamster cheek pouch served as a positive control. Within the GW-532 and GW-584 tumor cells, the hamster (red) and human (green) probes are identified in most cells and co-localized to the same cells (photoshop composite). On the other hand, human and hamster chromosomes show distinct, cell-specific localization within the human colon carcinoma xenograft, with hamster stromal tissue, and human colon carcinoma cells identified. DAPI background staining was performed on all tissues to identify the nuclei. Scale bar in the lower right corner corresponds to 100 µm for all GW-532 and GW-584 images and 67 µm for all of the higher magnification insets. For the GW-39 images, the scale bar corresponds to 200 µm.

The second and 25^th^ generation GW-532 transplants and the 20^th^ passage of GW-584 in the hamster cheek, as well as control GW-39 human colonic carcinoma growing in the hamster for over 40 years and proven to be totally human by cytogenetics [11], were probed for expression of human and hamster chromosomal materials. Hybridization of the control GW-39 human colonic carcinoma with human and hamster pancentromeric DNA depicted the tumor cells as having exclusively human pancentromeric genomic DNA, while adjacent hamster cheek pouch stromal cells showed the corresponding golden hamster centromeric DNA (Fig. 4). The GW-532 and GW-584 transplants, however, demonstrated signals for both the hamster and human probes within the same cells. These results establish that these cells are genetically human-hamster heterosynkaryons.

### PCR demonstration of human genes and EBV

A total of 24 human genes (listed in Table 1), as well as EBV, were examined by PCR. The human genes disclosed by PCR, *CD74, CXCR4, CD19, CD20, CD71, CD79b*, and vimentin (*VIM*), are normally located on human chromosomes 5, 2, 16, 11, 3, 17, and 10, respectively. However, as listed in Table 1, other genes on some of these same chromosomes were consistently absent. In GW-532, all were present in the primary tumor as well as in most of the 4 transplant generations examined (generations 2, 25, 39, and 115). In the GW-584 lymphoma, all were detected in the primary tumor and, except for *CD19* and *VIM*, also in all transplant passages examined (generations 1, 26, 28, and 57). Thus, these human genes were retained for over 33 months in continuous passages of GW-532, and for about 15 months for GW-584. It was noted that in several instances (e.g., *CD71* and *CD74*), the genes were not consistently detected within sequential tumor generations. Although there are several potential explanations, the most likely reasons would appear to be the quality of the specific gene DNA isolated from the individual tumor specimens, and/or the absolute quantity of human DNA within the isolate (ratio of human to hamster), Generational results listed as negative (i.e., undetectable), are not necessarily informative. However, a positive specific PCR reaction provides evidence for specific retention of the gene being probed.

**Table 1:** PCR detection of human genes in malignant hybrid xenografts and control tissues.

Gene	*CD74*	*CXCR4*	*CD19*	*CD20*	*CD71*	*CD79b*	*VIM*
Chromosome locus	5q32	2q22.1	16p11.2	11q12	3q29	17q23	10p13
**GW-532**							
Primary Tumor	+	+	+	+	ND	+	+
Gen-1	ND	ND	ND	ND	+	ND	ND
Gen-2	+	+	+	−	ND	+	+
Gen-19	ND	ND	ND	ND	−	ND	ND
Gen-25	+	−	+	+	+	+	+
Gen-39	+	+	+	+	ND	+	+
Gen-115	−	+	−	+	−	+	+
**GW-584**							
Primary Tumor	+	+	+	+	ND	+	+
Gen-1	+	+	−	+	+	+	−
Gen-20	+	+	−	+	−	+	−
Gen-28	+	+	−	+	ND	+	−
Gen-57	+	+	−	+	+	+	−
Raji Human Lymphoma	+	+	+	+	+	+	+
CHO Hamster Ovary	−	−	−	−	−	−	−

Human genes that were not detected in either GW-532 or GW-584 transplants			
Gene	Chromosome	Gene	Chromosome
*CD5*	11q12.2	*PLAGL2*	20q11.21
*CD22*	19q13.1	*CXCL12*	10q11.1
*CD23*	19p13.3	*MET*	7q31
*CD44*	11p13	*CDKN2A*	9p21.3
*CD30*	1p36	*TP53*	17p13
*CD80*	3q13.3	*TNC*	9q33.1
*CD86*	3q21	*GFAP*	17q21.31
*BCL-6*	3q27	*EGFR_VIII_*	7p11.2
*MIF*	22q11.23		

ND – Not Done due to limited quantity of DNA isolated from the tumor block.

**Figure 5:**
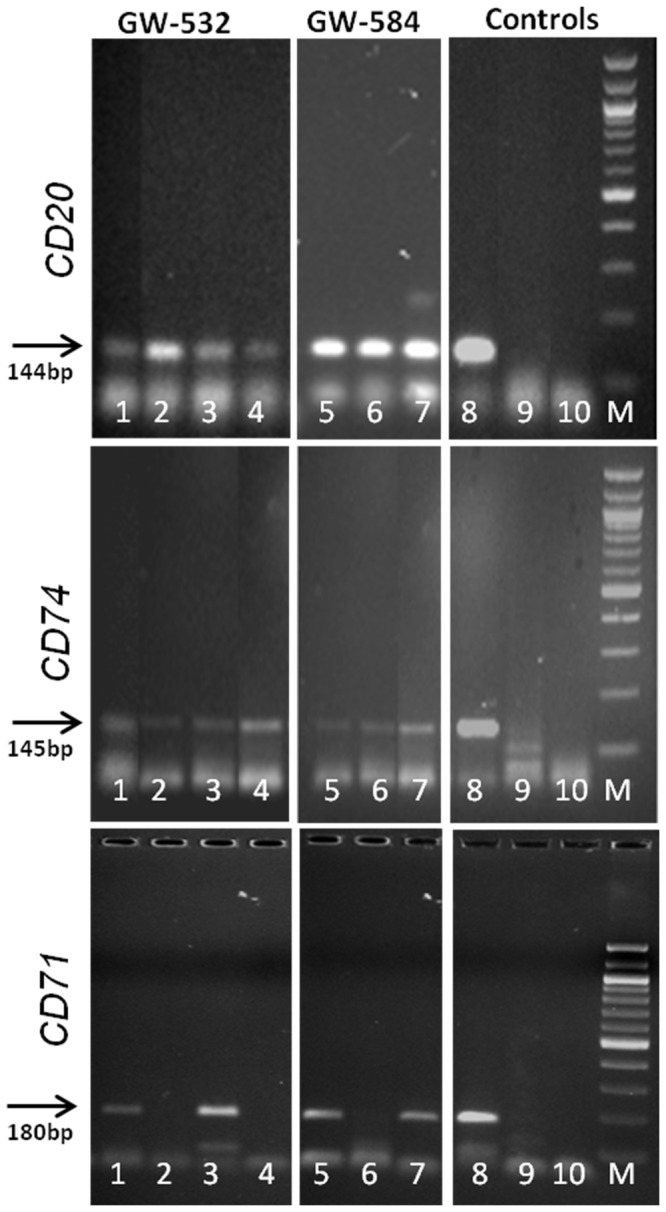
PCR evaluation of GW-532 and GW-584 for the presence of *CD20*, *CD74*, *and CD71* genes. DNA was extracted from formalin-fixed, paraffin-embedded specimens to be used as templates for PCR amplification with primers, as described in Supporting Information Table S1. The individual panels demonstrate the presence or absence of each gene within several generations of xenografted tumors. For PCR of *CD20*, the lanes contained GW-532 primary tumor (1), and xenograft tumors from generations 25 (2), 39 (3), and 115 (4); GW-584 xenografted tumors from generations 1 (5), 20 (6), and 57 (7); and controls Raji human lymphoma (8), CHO (Chinese hamster ovary) (9), and primers alone (10). Size markers are shown in lane M. Lanes are identical for PCR of *CD74*. For PCR of *CD71*, the lanes contained GW-532 xenografted tumors from generations 1 (1), 19 (2), 25 (3), and 115 (4); and GW-584 xenograft tumors from generations 1 (5), 20 (6), and 57 (7). Controls are identical to the *CD20* and *CD74* panels.

Examples of the PCR gels showing the presence of *CD20, CD74* and *CD71* in these tumors, with the appropriate positive (Raji lymphoma) and negative (CHO cells) controls, are presented in Fig. 5. The PCR results for EBV indicated that this viral DNA could not be detected in either of the two transplant tumors, yet showed positivity in control Raji Burkitt lymphoma cells (data not shown).

### IHC for human antigens

Twenty-four human antigens were examined for protein expression in the primary or transplanted tumors, as listed in Table S2 in the Supporting Information online. Only the proteins of CD74 (B cells, monocytes, and dendritic cells) and CD68 (histiocytes) were expressed in both primary tumors, while CD80 (granuloma cells) also was expressed in the original GW-584 specimen, but these and all others, including those most usually associated with B-cell neoplasms, were either not translated in any of the tumor transplants examined, or were below the threshold of staining despite using high concentrations of the detection antibodies, which could be related to denaturation of these proteins in the formalin-fixed, paraffin sections stored for many years. Since some antibodies show crossreactivity between human and rodent tissues, it was critical in the evaluation that any positivity in these tumors had to show a similar reaction in control human Raji lymphoma cells and absent in hamster spleen cells.

## Discussion

The heterotransplantation of human tumors to animal hosts has a long history, beginning with the use of immune privileged sites (brain, anterior chamber of the eye, hamster cheek pouch), and immunosuppressed hosts, including neonatal, immature rodents, athymic (nude) mice, and severe combined immunodeficiency (SCID) mice. During this time, the expectation has been that the human tumors grafted would retain morphological, biological, histogenetic, and biochemical properties, including the drug disposition of the patient, and thus be representative of the original neoplasm in the patient. As early as 1952, Greene suggested that the engraftment of the tumor in the golden hamster cheek pouch reflects its malignancy in the original patient [12], which has been again emphasized recently with human breast cancer transplants to the mammary fat pad of SCID mice [13]. Indeed, the growth of human tumor cells in recipient mice is used to define cancer-initiating, or stem-cell populations. These attributes rest on the thesis that the host is an in-vivo cell culture system, passively providing the environment to nourish and vascularize the human cancer cells in an ex-vivo controlled environment. Whereas early studies in immunosuppressed animals, immune privileged sites, or even immunodeficient nude mice rarely showed evidence of tumor dissemination and metastasis, with few exceptions, SCID mice are more amenable to xenogeneic tumor transplants metastasizing [13]–[15], so the immunological nature of the recipient host influences the biology of the transplant. Further, it is important to appreciate that established human cancer cell lines behave differently in such transplant models than primary human cancers, where the former usually have a higher engraftment rate and show more aggressive behavior than the latter [14], [16]. Of course, this also is influenced by the site of engraftment, whether subcutaneous, intravenous, orthotopic, or in a special location, such as the brain [16]. It should also be noted that, in general, hematopoietic tumor samples from patients have been more difficult to engraft in rodents than primary solid tumors [16], [17].

Hodgkin lymphoma cell lines have been transplanted successfully to the hamster cheek pouch, inducing tumors with as few as 100 cells in the inocula. The engrafted tumors were negative for EBV DNA. The transplanted cell lines had Hodgkin-Reed-Sternberg-like binucleate or multinucleate cells, which were replicated in these cultures and also observed in the transplants, resembling a “histiocytic lymphoma” or large cell lymphoma [18]. These tumor cell lines were proposed to show properties of a macrophage origin, consistent with the observations of Kaplan and Gartner with short-term cultures of giant cells from Hodgkin lymphoma [19]. However, as discussed below, more recent evidence points to a B-cell origin of Hodgkin cells [20]. Kapp et al. emphasized the difficulties in xenografting, but reported some success with Hodgkin lymphoma transplanted serially to the renal capsule of SCID mice, with a latency of appearance of the tumors of 3–5 months [14]. The tumors comprised activated B cells, since they stained for CD30 and CD20, and also had EBV transcripts in most of the HRS cells, although EBV also was present only in a few nonmalignant lymphoid bystander cells of the original patient specimens. The authors concluded that the transplanted tumor cells were either derived from EBV-superinfected HRS cells or from EBV-infected bystander cells, thus implicating neighboring tumor or other genetically-altered cells within the lymphomas. Indeed, the use of SCID mice for transplantation of human tumors, including lymphomas, has resulted in good grafting and growth that is similar to their clinical behavior in patients [13], [15].

However, the assumption of retention of fully human cells in animal xenografts, which is the basis of their use for predicting the behavior or properties of the original patient's tumor and the distinguishing property of stem cells, was first challenged by the observation that cell-cell fusion, with predominantly host properties, could result from xenografting to animal hosts, such as to the cheek pouch of unconditioned, adult hamsters [1], [7], [10]. This was observed by one of the authors in 15 different transplants of many different human cancer types, and the early transplants, usually already in the first engraftment, showed widespread metastasis and long-term propagation at various sites of the hamster [7], [10]. At that time, only karyological, immunological, and biochemical methods were used to define the transplants as human-hamster heterosynkaryons [1], [7], [21]–[23]. Recent advances now permit interrogating such tumors for their retention, transcription, and translation of human genes, and any possible relationship to the tumor of origin in the patient. The methods can be applied to formalin-fixed, paraffin-embedded sections to assess DNA of various genes and their translated proteins, so as to assess what genes have been retained, transcribed, and expressed in the transplants. FISH comprising both species genomes also can be performed on these paraffin sections, thus disclosing whether single cells are hybrid genetically (i.e., heterosynkaryons). Which stromal cells (e.g., fibroblasts or macrophages) of the hamster participated in these fusions is not known.

Our findings demonstrate the genetic transfer of the malignant and metastatic phenotypes, and other human gene functions, from two human lymphomas to the stroma of their xenogeneic host by cell-cell fusion, resulting in malignant heterosynkaryons. The first evidence of spontaneous metastasis, and presumably spontaneous fusion, was observed within 17–21 days of the initial cheek-pouch transplant. Indeed, we reported a similar experience recently, whereby a human glioblastoma multiforme (GBM) transplant that had human and hamster chromosomes in the same malignant cells, which were metastatic in hamsters, retained human and hamster genomic DNA (heterosynkaryons) after spontaneous cell-cell fusion in vivo [10]. This confirmed their propagation as interspecies hybrids, but also the transcription and translation of several human genes associated with GBM, including a morphological growth pattern of the original tumor in these transplants [10]. In this GBM model, at least 7 human genes (of 12 tested) were retained, with at least 3 human gene products being expressed during long-term passage in hamsters, while the GBM's morphology and highly metastatic phenotype also were sustained [10]. Prior studies have shown that cell-cell fusion can occur spontaneously in vivo between human tumor and normal rodent host cells [10], [24], [25], yet the question of how stable these tumors are for continued expression of their parental genomes, and any specific genes, has not been addressed prior to our recent experience with the GBM transplant and, now, these two Hodgkin lymphoma-derived xenografts. Not only do these lymphoid tumors show similar highly malignant features as the GBM, thus confirming this process in two different cell types xenografted, but they displayed appropriate morphological differentiation by having similar lymphoid features to the original human Hodgkin malignancies grafted. Other studies of cell fusion have elucidated organ-specific metastatic gene signatures [26], supporting our view that genetic transfer of organoid features also is likely.

Whereas the genes expressed in the GBM transplants included *CD74, CXCR4, PLAGL2, GFAP, VIM, EGFR*, and *TP53*, where 5 (*CD74, CXCR4, PLAGL2, GFAP*, and *EGFR*), or more, are associated with GBM, the 2 lymphomas hybridized with hamster cells, GW-532 and GW-584, retained, respectively, three (*CD20*, *CD79b, CD19*) and two (*CD20*, *CD79b*) genes that are restricted to B-cell lineage, or genes associated with high proliferation (*CD71* and *CD74*). It is noteworthy that both the GBM and lymphoma hybrid transplants all retained *CD74*, *CXCR4* and *VIM* genes, suggesting that these are important to evaluate in other examples of spontaneous fusions of human tumor and rodent host cells. In the GBM transplant line, 3 of the genes (*CD74, CXCR4*, and *PLAGL2*) showed translation to their respective proteins by IHC, whereas no genes were translated in the GW-532 and GW-584 transplants, at least at the detection sensitivity of the IHC used. This is either because their translation was blocked or the age or processing of the sections did not permit disclosure of these proteins by IHC. However, stromal cells within the patients' primary tumors did stain for CD68, CD74 or CD80, indicating that these sections did express these proteins despite their age being similar to that of the transplants. Further, the xenograft sections did stain for actin when tested with various antibodies, although the reaction was not specific for human cells (results not shown). Although CD80 protein expression within the primary tumor's granuloma may appear inconsistent with the observation that the *CD80* gene was not detected in this same specimen, it should be noted there are significant differences in the sensitivity of the two methodologies. Immunohistochemistry can detect single-cell positive reactions, whereas PCR requires extraction of the whole tissue with potential for dilution of the specific gene below the levels required for detection.

Finally, FISH studies confirmed that the fused cells were heterosynkaryons, since various cell populations had both human and hamster genomic DNA signals present in the same malignant cells. It is also noteworthy that 3 of the 7 chromosomes with demonstrated retention of human genes in these lymphoma transplants (i.e., chromosomes 2, 5 and 17) are among those implicated as having genetic gains in classical Hodgkin lymphoma [27]–[30]. We hypothesize that, similar to in-vitro cell fusion, in-vivo hybridization may segregate genes that propagate signatures of the original donor tumors.

It is interesting to speculate what these human lymphoma-hamster hybrid cell lines may reveal with regard to the pathology and lineage characteristics of the original human neoplasms. Both original tumors showed predominant features of Hodgkin lymphoma, which are retained in their transplant lines, GW-532 and GW-584, during long-term xenogeneic passage for up to 5 and 6 years, respectively, in addition to the retention of 7 human genes of 24 tested.

An important finding is that the hybrid lymphoid tumor transplants also retained morphological features of the original human Hodgkin lymphoma specimens, as did the GBM transplant for glioma features [10]. Even after years of propagation in vivo, both lymphoid tumors showed a similar, somewhat uniform, undifferentiated, mitotic-rich, appearance with occasional giant cells resembling HRS cells, both in the cheek pouch grafts and their spontaneous metastases to major organs. The first evidence of widespread metastases was observed within 2 weeks of the very first transplants to the hamster cheek pouch, and continued this aggressive behavior for the 5 to 6 years that these lymphoma lines were maintained in hamsters. Hence, despite becoming hamster-human heterosynkaryons showing rapid growth and spread in normal adult hamsters, presumably losing much of the human genome, they retained the phenotypic characteristics of their lymphoma derivation and B-cell genetic signature. EBV DNA was not detected in the original tumor specimens or in the transplants, yet virus-like particles resembling those depicted in human leukemias and lymphomas were observed by electron microscopy in the original and transplanted GW-532 tumors, including metastases [9]. Kaplan et al. also observed C-type viruses in the human lymphoma cell line transplanted [31].

The fate and role of Hodgkin lymphoma cells, particularly the proliferation of HRS cells, has been investigated since the initial establishment of in-vitro cell lines and also transplants to hamsters and mice [18], [32]. There is a general consensus that Hodgkin lymphoma and HRS cells are of B-cell lineage [20], although a macrophage derivation also has been proposed [18], [19]. Macrophages, as defined by CD68 staining, were prominent in the original GW-532 specimen, but the *CD68* gene was not retained or expressed in the heterotransplants. CD68 was not expressed, as tested by IHC, in the HRS cells of the original biopsy specimens.

In none of the Hodgkin lymphoma transplant studies reported over the past 30+ years has a suggestion been raised that the xenografts changed their morphology or genetics, even when metastases were observed in SCID mice. However, most studies examined short-term cultures or established cell lines, and only relatively few passages of these cell lines in vivo; very rarely were primary human lymphomas grafted continuously in animal hosts.

HRS cells, which are considered to be the malignant cell population in Hodgkin lymphoma, usually do not express immunoglobulin or other B-cell markers, in contrast to other B-cell malignancies [33]–[35]. It has been suggested that functional immunoglobulin and other B-cell genes are not transcribed, presumably as a result of epigenetic silencing or other mechanisms [36]. However, our two transplant lines did show retention of B-cell genes as well as other human genes associated with proliferation and malignancy. Further, Newcom et al. reported that the L428 Hodgkin lymphoma cell line, while consisting mostly of HRS cells, also had a population of phenotypic B cells that appeared to generate the HRS cells [37]. It was later shown that the same cell line had B-cell subpopulations that also were found in the blood of newly diagnosed Hodgkin lymphoma patients, suggesting that these may be the initiating cells for this disease [38]. Since the heterosynkaryons of human Hodgkin lymphoma and normal hamster host cells, which showed the continuous propagation of lymphoid tumors with a few human genes, retained the B-cell-restricted genes (*CD19, CD20*, and *CD79b*) within the predominant neoplastic population, these clonotypic B cells may be the initiating- or stem-cells of Hodgkin lymphoma. Indeed, Jones et al. considered HRS cells to be terminally differentiated, and therefore not the cells generating Hodgkin lymphoma, claiming that B cells are the origin of HRS cells, as supported by the presence of CD20^+^ clonotypic B cells [38]. Hence, the method of somatic cell fusion, which has been used to map chromosomes and genes [39], and to analyze tumor promoter or suppressor chromosomes or genes [40], [41], in the in-vivo setting may also elucidate genes and cell populations critical to oncogenicity, heterogeneity, metastasizability, and organoid differentiation.

In addition to the mechanism of cell-cell fusion inducing the horizontal transmission of malignancy, one of us reported already in 1981 the in-vivo horizontal transmission of malignancy from human tumor transplants to recipient nude mouse cells, resulting in tumors of fibrosarcomatous appearance [42]. This confirmed the earlier observation of Ehrlich and Apolant, at the beginning of the last century, that carcinomas can induce sarcomas in normal mice [43]. More recent studies have indeed confirmed that malignancy can be transferred to normal cells by DNA in exosomes (or microvesicles) [3] or from sera [8], [44] of cancer patients. Hence, mechanisms beyond cell-cell fusion can transfer oncogenic DNA to normal cells, resulting in the horizontal transmission of malignancy. In the studies reported here, however, other genes of the neoplasm also were sustained in the malignant progeny cultivated for up to 6 years in a new recipient host species. As in our experience with a transplanted human glioblastoma multiforme [10], we can only speculate how these or other genes (not revealed, but possibly present) contributed to retention of the original tumor's morphology after heterotransplantation and heterosynkaryon formation. Intriguing is the finding, as mentioned, that *CD74*, *CXCR4* and *VIM* human genes are common to both the glioblastoma and these two lymphoma transplants, while the others transcribed in each neoplastic type are associated with their particular organ-specific phenotype (*PLAGL2, GFAP*, and *EGFR* in glioblastoma and *CD19, CD20*, and *CD79b* in the two Hodgkin lymphomas).

The interactions between malignant cells and their surrounding stromal cells for understanding both oncogenesis and metastasis has been the subject of intense investigation in recent years, but the molecular basis at various levels is still unknown. Stromal fibroblasts clearly play a role in cancer initiation and progression [45]. Genetic alterations of adjacent stromal cells of recipient hosts have been reported after xenografting human tumors [46], being both similar and different from those in the tumor [47].

In conclusion, these transplantation studies with two human Hodgkin lymphomas confirm the recent observations with a human glioblastoma [10] that spontaneous interspecies hybridization resulted in serial propagation of heterosynkaryons that retained select human genes consistent with their original cell type, a highly metastatic behavior, and morphological resemblances to the original tumor specimens, but in this case a B-cell signature in contrast to glioblastoma-related genes present in the GBM/hamster hybrid tumor. Interestingly, this event probably occurred within the first 2–3 weeks of the initial transplant, making the involvement of endogenous hamster viruses highly unlikely. Whether in-vivo cell-cell fusion is a prominent mechanism or just one of several resulting in the horizontal transmission of malignant and organoid genes to neighboring cells of the stroma deserves further study. It should be noted that evidence of in-vivo cell fusion in patients has been claimed on the basis of premature chromosome condensation in tumors [48] and the disclosure of myeloma-osteoclast hybrids [49]. We suggest that the transfer of malignant DNA to adjacent stromal cells may be more relevant to tumor heterogeneity and progression than has been appreciated.

## Supporting Information

Table S1 **Primer sequences employed for PCR detection of human genes.**
(DOC)

Table S2 **Human proteins tested on the GW-532 and GW-584 lymphomas by immunohistochemistry of primary and/or xenograft tissues.**
(DOC)
