# Supplementary material for: Horizontal Transmission of Malignancy: In-Vivo Fusion of Human Lymphomas with Hamster Stroma Produces Tumors Retaining Human Genes and Lymphoid Pathology
Source: PLoS One. 2013 Feb 6;8(2):e55324. doi: 10.1371/journal.pone.0055324 (PMC3566191; doi:10.1371/journal.pone.0055324)
Supplement: Table S1 — Primer sequences employed for PCR detection of human genes. (DOC) [file pone.0055324.s001.doc]

Table S1. Primer sequences employed for PCR detection of human genes.

| ***Gene*** | **Chromosome** | **Forward primer** | **Reverse primer** | **PCR (bp)** | **Source** |
| --- | --- | --- | --- | --- | --- |
| ***CD74*** | ***5*** | **GCCCTGTACACAGGCTTTTCCATCC** | **TCATGCGCAGGTTCTCCAGC** | **145** | **Vector NTI** |
| ***CRCX4*** | ***2*** | **CCGCTTCTACCCCAATGACT** | **GGCGAAGAAAGCCAGGAT** | **190** | **UniSTS 040008** |
| ***PLAGL2*** | ***20*** | **CCTTCCCTTTTTGGTAGCTGTTT** | **TTATGGTGAACACCCTTTTCCCT** | **304** | **UniSTS 136576** |
| ***GFAP*** | ***17*** | **GCCTTAGAGGGGAGAGGAG** | **AGGGCTGACACGTCCAC** | **132** | **UniSTS 49186** |
| ***TNC*** | ***9*** | **GCCTCACCTCCTCTGTGATT** | **AAAAAGGGATGGCTTCCAAT** | **271** | **UniSTS 48958** |
| ***MIF*** | ***22*** | **CACCAGAAGGTTGGGGTG** | **ACTATTACGACATGAACGCGGT** | **147** | **UniSTS 24485** |
| ***CXCL12*** | ***10*** | **CTGGGCAAAGCCTAGTGAAG** | **CTCCCAGAAGAGGCAGACCT** | **113-114** | **UniSTS 38915** |
| ***VIM*** | ***10*** | **CCGACACTCCTACAAGATTTAGA** | **CAAAGATTTATTGAAGCAGAACC** | **143** | **UniSTS 32309** |
| ***CDKN2A*** | ***9*** | **CATCCCCGATTGAAAGAACC** | **AATGGACATTTACGGTAGTGGG** | **204** | **UniSTS 27844** |
| ***TP53*** | ***17*** | **GTCCTGCTTGCTTACCTCGCTTAGT** | **ACCTGATTTCCTTACTGCCTCTTGC** | **200** | **UniSTS 273402** |
| ***EGFRvIII*** | ***7*** | **GAGTCGGGCTCTGGAGGAAAAG** | **CCACAGGCTCGGACGCAC** | **352** | **a** |
| ***cMET*** | ***7*** | **GCCAGATGAAATACTTCC** | **AAGACCAAAATCAGCAACC** | **175** | **UniSTS:48429** |
| ***CD5*** | ***11*** | **CAGTGAAATCGGCTTTCTCC** | **GGGGAGTCATAAGACAGAGGG** | **343-344** | **UniSTS:27058** |
| ***CD19*** | ***16*** | **CAAGTCCCCAAGATTCACAC** | **AGGAATACAAAGGGGACTGG** | **174** | **UniSTS 23292** |
| ***CD20*** | ***11*** | **CTGAGGATCCAAGGCCTCAAATCTC** | **ACCGGATCCCTATCAGCGATTTCAT** | **144** | **UniSTS:38061** |
| ***CD22*** | ***19*** | **CGAGTTTCCCCAGACACC** | **TCTGGGTAGCAAGGAGAGTG** | **196** | **UniSTS:34464** |
| ***CD23*** | ***19*** | **GAGCAAGACCCTGAAGACCC** | **TGGAGAGGGTGCTGTTGG** | **224** | **UniSTS:44277** |
| ***CD44*** | ***11*** | **GCAGAATGTGGACATGAAGA** | **ATGCTAAAAAAGATTCGCAATG** | **150** | **UniSTS:47355** |
| ***CD79b*** | ***17*** | **TCCGAAGCAGTCACTGAGG** | **TGAGGACATAGTGACGCTGC** | **128** | **UniSTS:90981** |
| ***CD30*** | ***6*** | **ACAGGGCTTAGAAACTGACTGC** | **AGGAAGGTCATGTTCTTTCAGC** | **158** | **UniSTS:8024** |
| ***CD71*** | ***3*** | **ATGGGAACTGCCTCTTTCCT** | **GGAGAAGGTCTTTCAACCTGG** | **180** | **UniSTS:19879** |
| ***CD80*** | ***3*** | **TAACAGTGTCCGCAGAAGCA** | **TGACAAGACAATTCAAGATGGC** | **277** | **UniSTS:60447** |
| ***CD86*** | ***3*** | **ATTATCCACCAAAGCTGTTCAGG** | **AATGTGGCTGTGCAATACATGAC** | **349** | **UniSTS:172908** |
| ***EBV*** |  | **GCCAGAGGTAAGTGGACTTT** | **TGGAGAGGTCAGGTTACTTA** | **241** | **b** |
| ***BCL6*** | ***3*** | **CGCAGAATGGGATGAGACAA** | **GTGTCCTCACGGTGCCTTTT** | **146** | **UniSTS:34563** |

a: Rae et al. (2004). EGFR and EGFRvIII expression in primary breast cancer and cell lines. Breast Cancer Res Treat 87: 87–95.

b: Chiou et al. (2005). Discovery of Epstein–Barr virus (EBV)-encoded RNA signal and EBV nuclear antigen leader protein DNA sequence in pet dogs. J Gen Virology 86: 899–905.
