# Supplementary material for: Horizontal Transmission of Malignancy: In-Vivo Fusion of Human Lymphomas with Hamster Stroma Produces Tumors Retaining Human Genes and Lymphoid Pathology
Source: PLoS One. 2013 Feb 6;8(2):e55324. doi: 10.1371/journal.pone.0055324 (PMC3566191; doi:10.1371/journal.pone.0055324)
Supplement: Table S2 — Human proteins tested on the GW-532 and GW-584 lymphomas by immunohistochemistry of primary and/or xenograft tissues. (DOC) [file pone.0055324.s002.doc]

| **Table S2. Human proteins tested on the GW-532 and GW-584 lymphomas** **by immunohistochemistry of primary and/or xenograft tissues.** | | | |
| --- | --- | --- | --- |
| **Protein** | **Ab clone** | **Source** | **Comments** |
| CD74 | LL1 | 1 | Primary tumors were positive;  xenografts were negative |
| CD3 | A0452 | 2 | Primary tumors – background T-cells were positive; xenografts were negative |
| CD5 | UCH-T2 | 3 |  |
| CD15 | 347420 | 4 |  |
| CD19 | LE-CD19 | 2 |  |
| CD20 | LL1 | 1 |  |
| CD22 | A20 | 1 |  |
| CD23 | M-L233 | 3 |  |
| CD30 | M0751 | 2 |  |
| CD68 | M814 | 2 | Primary tumors – background histiocytes were positive; xenografts were negative |
| CD71 | 1E6 | 5 |  |
| CD79a | JCB117 | 2 |  |
| CD79b | ZL9-3 | 3 |  |
| CD80 | 2A2 | 5 | Primary GW-584 was positive; Primary GW-532 was negative; all xenografts were negative |
| DR | L243 | 1 |  |
| PLAGL2 | C-16 | 3 |  |
| Vimentin | V9 | 2 |  |
| CXCR4 | 12G5 | 3 |  |
| CXCL12 | HBA-72 | 3 |  |
| MIF | Q18 | 3 |  |
| EGFR | 0.N.268 | 6 |  |
| TROP2 | AF650 | 5 |  |
| FUT4 | HI98 | 5 |  |
| Tenascin | T245 | 3 |  |
| Sources: 1-Immunomedics, Inc. (Morris Plains, NJ); 2-Dako (Carpinteria, CA); 3-Santa Cruz Biotechnology, Inc. (Santa Cruz, CA); 4-Becton Dickinson (Franklin Lakes, NJ); 5-LifeSpan Biosciences, Inc. (Seattle, WA); 6-R&D Systems (Minneapolis, MN); | | | |
